# Golgi-Related Proteins GOLPH2 (GP73/GOLM1) and GOLPH3 (GOPP1/MIDAS) in Cutaneous Melanoma: Patterns of Expression and Prognostic Significance

**DOI:** 10.3390/ijms17101619

**Published:** 2016-10-01

**Authors:** Piotr Donizy, Maciej Kaczorowski, Przemyslaw Biecek, Agnieszka Halon, Teresa Szkudlarek, Rafal Matkowski

**Affiliations:** 1 Department of Pathomorphology and Oncological Cytology, Wroclaw Medical University, Borowska 213, 50-556 Wroclaw, Poland; octopuso@wp.pl (M.K.); ahalon2@gmail.com (A.H.); tszkudlarek@poczta.fm (T.S.); 2 Faculty of Mathematics and Information Science, Warsaw University of Technology, Koszykowa 75, 00-662 Warsaw, Poland; przemyslaw.biecek@gmail.com; 3 Department of Oncology, Wroclaw Medical University; pl. Hirszfelda 12, 53-413 Wroclaw, Poland; rem@onet.pl; 4 Lower Silesian Cancer Center, Hirszfelda 12, 53-413 Wroclaw, Poland

**Keywords:** GOLPH2, GOLPH3, melanoma, tumor-associated macrophages, cancer-associated fibroblasts

## Abstract

GOLPH2 and GOLPH3 are Golgi-related proteins associated with aggressiveness and progression of a number of cancers. Their prognostic significance in melanoma has not yet been analyzed. We performed immunohistochemical analysis for GOLPH2 and GOLPH3 in 20 normal skin, 30 benign nevi and 100 primary melanoma tissue samples and evaluated their expression in three compartments: cancer cells, tumor-associated macrophages (TAMs) and cancer-associated fibroblasts (CAFs). High levels of both proteins in melanoma cells were associated with characteristics of aggressive disease, and shorter disease-free survival (DFS) and cancer-specific overall survival (CSOS). On the contrary, increased numbers of GOLPH2-positive and GOLPH3-positive TAMs were observed in thinner, non-ulcerated tumors, with brisk lymphocytic reaction and absent lymphangioinvasion. Distant metastases were not observed among patients with high numbers of GOLPH2-positive TAMs. Increased expression of either protein in TAMs was related to prolonged CSOS and DFS. Similarly, GOLPH3-expressing CAFs were more frequent in thin melanomas with low mitotic rate, without ulceration and lymphangioinvasion. Moreover, increased GOLPH3-positive CAFs correlated with the absence of regional or distant metastases, and with longer CSOS and DFS. GOLPH2 expression was not observed in CAFs. Our results suggest that GOLPH2 and GOLPH3 play a role in melanoma progression and are potential targets for molecular-based therapies.

## 1. Introduction

Golgi apparatus is a multifunctional organelle described for the first time over 100 years ago. Its best characterized functions involve modification of proteins and lipids, their sorting and dispatching in transport vesicles to various intracellular destinations or for exocytosis. Given the central role of Golgi for various cell processes, its dysregulation is a likely feature in carcinogenesis. However, alterations of Golgi function in cancer are still insufficiently studied.

GOLPH2 (Golgi phosphoprotein 2, also named GP73 and GOLM1) is a resident 73 kDa Golgi membrane glycoprotein expressed chiefly in epithelial lineage of cells [1]. While the physiological role of GOLPH2 remains vague, its upregulated expression was observed in a number of diseases. Initially, GOLPH2 upregulation was reported in neoplastic and non-neoplastic liver pathologies, and a number of studies postulated its serum level as a novel marker of hepatocellular carcinoma [2,3,4,5,6,7,8]. Subsequently, enhanced GOLPH2 expression was reported in other cancer settings [9,10,11,12,13,14,15]. To the best of our knowledge, GOLPH2 expression in melanoma patients has not been previously analyzed.

GOLPH3 (Golgi phosphoprotein 3, also named GOPP1/MIDAS), a highly conserved 34 kDa protein localized mostly in the trans-Golgi network, provides numerous cellular functions [16]. Binding of PI4P and MYO18A by GOLPH3 links Golgi membrane to F-actin cytoskeleton and is essential for vesicular trafficking [17,18]. As a side effect of these interactions, Golgi apparatus adopts a characteristic extended ribbon structure [18]. GOLPH3 also plays a role in mitochondrial biogenesis [19,20], cell adhesion and migration [21,22,23], cytokinesis [24], and promotes Golgi dispersal and survival response after DNA damage [25]. Its regulatory contribution to protein glycosylation in the Golgi may affect a wide range of cell functions [16]. A recent study established that GOLPH3 has oncogenic properties mediated via mTOR signaling, and that GOLPH3 gene at 5p13 region is frequently amplified in a number of cancers [26]. Close association between GOLPH3 expression and patient survival in a range of neoplasms (a meta-analysis in [27]) make the protein a potential target of new therapies and warrant further investigations. We believe that no clinicopathological analysis of GOLPH3 expression in tissue specimens from patients with cutaneous melanoma has been published so far.

Tumor stroma has long been known to be a relevant and active player in carcinogenesis. Accumulating evidence shows that cancer-associated fibroblasts (CAFs) and tumor-associated macrophages (TAMs) representing key non-malignant constituents of melanoma microenvironment, strongly influence cancer growth and progression [28,29]. Secretion of growth factors, modulation of local immune response, regulation of angiogenesis, remodeling of extracellular matrix by secreted metalloproteinases are some of the mechanisms involved [28,29]. Moreover, recent studies demonstrate that fibroblasts and macrophages play a critical role in treatment resistance in melanoma [30,31]. Considering the distinct position of melanoma-associated stromal cells during carcinogenesis and a complexity of interactions between them and malignant melanocytes, we decided to individually examine neoplastic and stromal components. There is no published analysis of GOLPH2 and 3 expression in melanoma’s stroma and available studies in other malignancies lack a systematical assessment of these proteins in a non-neoplastic tumor microenvironment.

We performed immunohistochemical analysis for GOLPH2 and GOLPH3 and evaluated their expression in three compartments: cancer cells, TAMs and CAFs. This is the first immunohistochemical analysis of GOLPH2 and 3 expression in a clinical melanoma cohort, and the first one addressing the significance of GOLPH2 and GOLPH3 expression in the stromal components. The goal of our study was to examine the relationship of GOLPH2 and GOLPH3 expression levels in different compartments of primary melanoma tissue samples with other histopathological and clinicopathological parameters, especially with patient survival.

## 2. Results

### 2.1. Expression of GOLPH2 and GOLPH3 in Normal Skin and Benign Nevi

**Figure 1:**
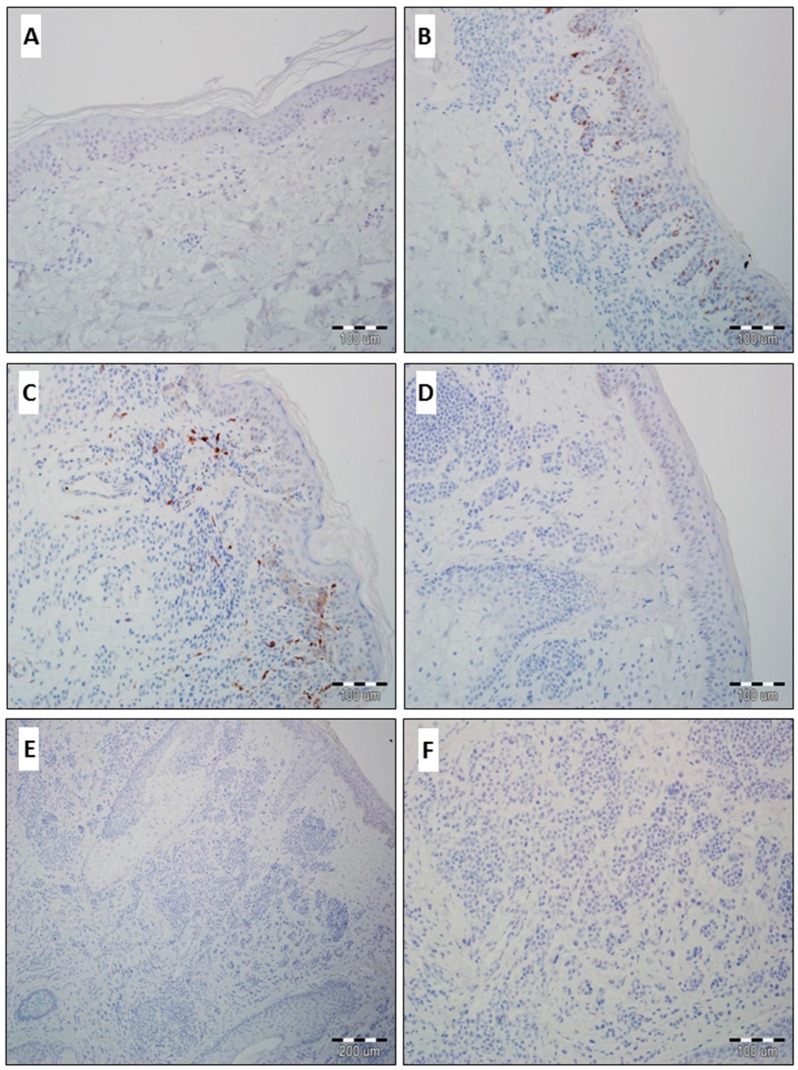
Normal skin without GOLPH3 immunoreactivity ((**A**) 200×, hematoxylin); Scanty expression of GOLPH2 in a compound skin nevus ((**B**) 200×, hematoxylin); Scanty expression of GOLPH3 in a compound skin nevus ((**C**) focal and low GOLPH3 reactivity in benign melanocytes and a small percentage of GOLPH3-positive stromal cells; 200×, hematoxylin); Lack of GOLPH2 expression in an intradermal nevus ((**D**) 200×, hematoxylin); Lack of GOLPH3 expression in an intradermal nevus ((**E**), 100×; (**F**), 200×, hematoxylin).

In all cases of normal skin, the epidermis was negative for GOLPH2 and GOLPH3 (Figure 1A). We observed predominantly similar results in benign skin nevi. Only in four cases (all were diagnosed as compound benign skin nevus) scanty immunoexpression of GOLPH2 and GOLPH3 in benign melanocytes and stromal compartment of the nevi was observed (Figure 1B,C). Other benign nevi were negative (Figure 1D–F).

### 2.2. Expression of GOLPH2 in Melanoma Cells and Tumor-Associated Macrophages (TAMs)

The expression of analyzed proteins in evaluated compartments was divided into two levels based on IRS score for melanoma cells (low: <6 vs. high: ≥6), the number of GOLPH2- and GOLPH3-positive macrophages (<20 vs. ≥20) and the number of GOLPH3-positive fibroblasts (<10 vs. ≥10). The IRS scale for melanoma cells assigns a score for the percentage of cells demonstrating reaction (0–4 points) and for reaction intensity (0–3 points). The IRS score is the product of the scores for these two parameters (0–12 points). A detailed description of pathological assessment of protein expression is included in the Material and Methods section.

**Figure 2:**
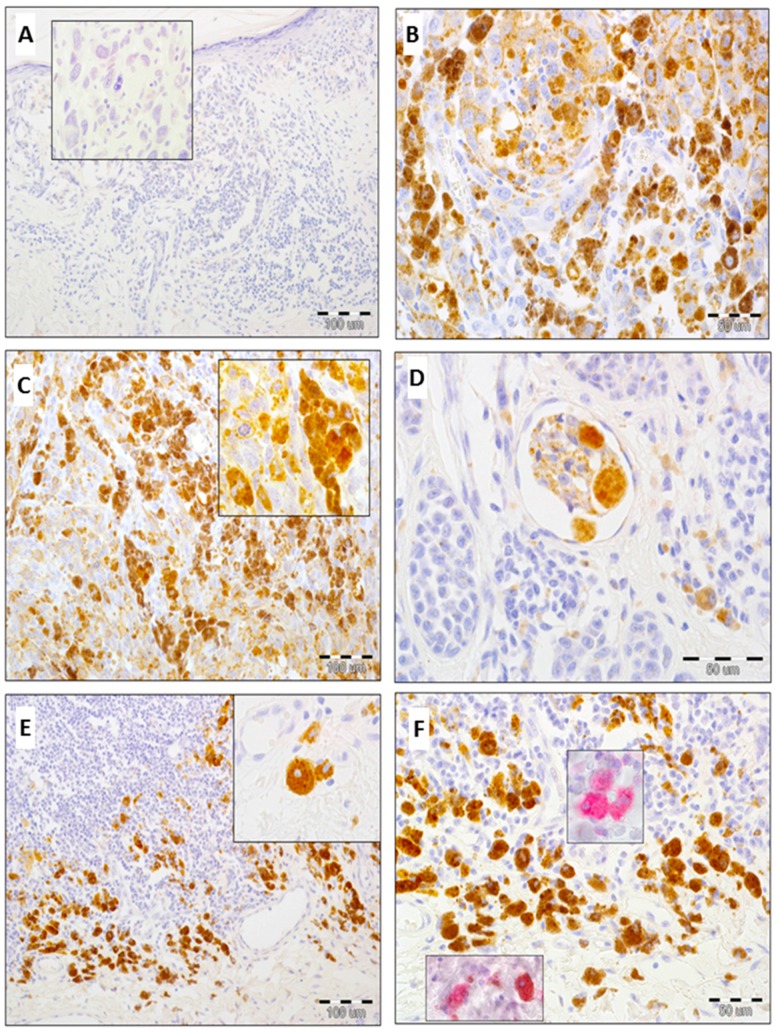
Immunohistochemical analysis of GOLPH2 expression in skin melanoma. Lack of GOLPH2 immunoreactivity in melanoma cells, higher magnification: pathologic mitosis with no GOLPH2 expression ((**A**) 100×, 600×, hematoxylin); High cytoplasmic expression of GOLPH2 in neoplastic melanocytes ((**B**) 200×, hematoxylin, (**C**) 100×, 600×, hematoxylin); Lymphangioinvasion with a nest of GOLPH2-positive melanoma cells ((**D**) 400×, hematoxylin); Strong GOLPH2 expression in tumor-associated macrophages, higher magnification: round cell with a macrophage phenotype, low nuclear/cytoplasmic ratio and strong cytoplasmic GOLPH2 reactivity ((**E**) 100×, 600×, hematoxylin); Strong TIL (tumor-infiltrating lymphocytes) infiltration (negative for GOLPH2) at the base of tumor mass and enhanced expression of GOLPH2 in macrophages, higher magnification: immunohistochemical reaction (red chromogen) with anti-CD68 antibody ((**F**) 200×, 600×, hematoxylin).

High expression of GOLPH2 in cancer cells was detected in 31% of melanomas (31/100), whereas low/absent expression was found in 69% of cases (69/100). No GOLPH2 expression in melanoma cells was observed in 29 primary tumors (Figure 2A–D). Mean IRS score for malignant melanocytes was 3.27 ± 3.1 (median: 2).

Upregulated expression of GOLPH2 in TAMs was observed in 63% of cases (63/100), low/absent expression in 37% of melanomas (37/100). 23 primary tumors presented no GOLPH2 immunoreactivity in TAMs (Figure 2E,F).

### 2.3. Expression of GOLPH3 in Melanoma Cells, Tumor-Associated Macrophages (TAMs) and Cancer-Associated Fibroblasts (CAFs)

**Figure 3:**
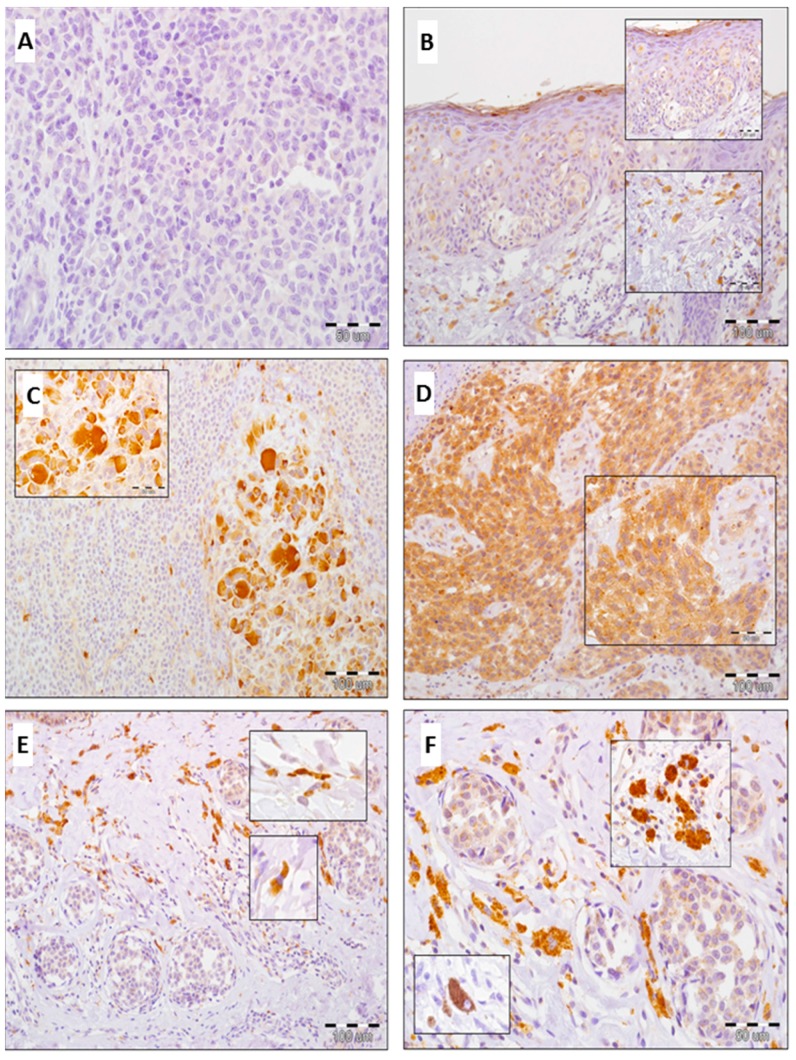
GOLPH3 expression in skin melanoma patients. Lack of GOLPH3 reactivity in invasive melanoma ((**A**) 400×, hematoxylin); In situ component of melanoma with scanty expression of GOLPH3 in neoplastic cells and with moderate reaction in macrophages and fibroblasts ((**B**) 100×, 200×, 400×, hematoxylin); High cytoplasmic GOLPH3 expression in neoplastic melanocytes and lack of GOLPH3 reactivity in TILs, higher magnification: bizarre neoplastic melanocytes with typical malignant cytomorphology (hyperchromasia and aberrant nuclear shape, high nuclear/cytoplasmic ratio, prominent nucleoli) ((**C**) 100×, 600×, hematoxylin); Diffuse, strong GOLPH3 reactivity in melanoma cells, higher magnification: GOLPH3-positive melanoma cells with atypical mitotic figures ((**D**) 100×, 400×, hematoxylin); High percentage of GOLPH3-positive fibroblasts in stromal compartment of melanoma, higher magnification: typical morphology of GOLPH3-positive fibroblasts (elongated shape, adequate nuclear/cytoplasmic ratio, inconspicuous nucleoli) ((**E**) 100×, 600×, hematoxylin); Tumor-associated macrophages with strong expression of GOLPH3, higher magnification: typical morphology of macrophages (predominantly oval shape without morphological features of malignancy, positive anti-CD68 reaction) ((**F**) 200×, 600×, hematoxylin).

Elevated level of GOLPH3 expression in melanoma cells was detected in 51% of cases (51/100), whereas low/absent expression in 49% of cases (49/100) (Figure 3A–D). Mean IRS score for melanoma cells was 5.16 ± 3.0 (median: 6).

GOLPH3 expression in TAMs and CAFs was upregulated in 68% and 78% primary tumors, respectively. Low/absent expression of GOLPH3 in TAMs and CAFs was demonstrated in 32% and 22% primary tumors, respectively (Figure 3E,F).

### 2.4. Analysis of Correlations between GOLPH2 and GOLPH3 Immunoreactivity in Melanoma Cells and Clinicopathological Parameters

Increased immunoreactivity of GOLPH2 and GOLPH3 in melanoma cells was statistically linked with thicker tumors, regional lymph node metastases (only SLN metastases for GOLPH3), locoregional recurrence, lymphangioinvasion, presence of ulceration, higher mitotic rate, and lower TIL (tumor-infiltrating lymphocytes) grade (Table 1, Table 2, Table 3 and Table 4). Overexpression of GOLPH3 was more frequently observed in nodular and acral-lentiginous melanomas (Table 4).

### 2.5. Analysis of Correlations Between GOLPH2 and GOLPH3 Immunoreactivity in Stromal Compartment and Clinicopathological Parameters

Increased numbers of GOLPH2-positive and GOLPH3-positive macrophages correlated with thinner tumors, absence of ulceration, brisk TILs, lack of lymphangioinvasion and lower locoregional recurrence rate (Table 1, Table 2, Table 3 and Table 4). Moreover, increased GOLPH3-positive macrophages were related to low proliferative activity of cancer cells and characterized tumors localized on the trunk and extremities. (Table 3 and Table 4). Distant metastases were not observed among patients with high numbers of GOLPH2-positive TAMs (Table 1).

Increased GOLPH3-positive fibroblasts were observed in thinner, non-ulcerated melanomas, with low mitotic rate and without lymphangioinvasion. Moreover, high numbers of CAFs correlated with absence of regional and distant metastases (Table 3 and Table 4).

### 2.6. Impact of Golgi-Related Protein Expression in Neoplastic Cells and Stromal Compartment on Melanoma Patient Survival—Kaplan-Meier Analysis

**Figure 4:**
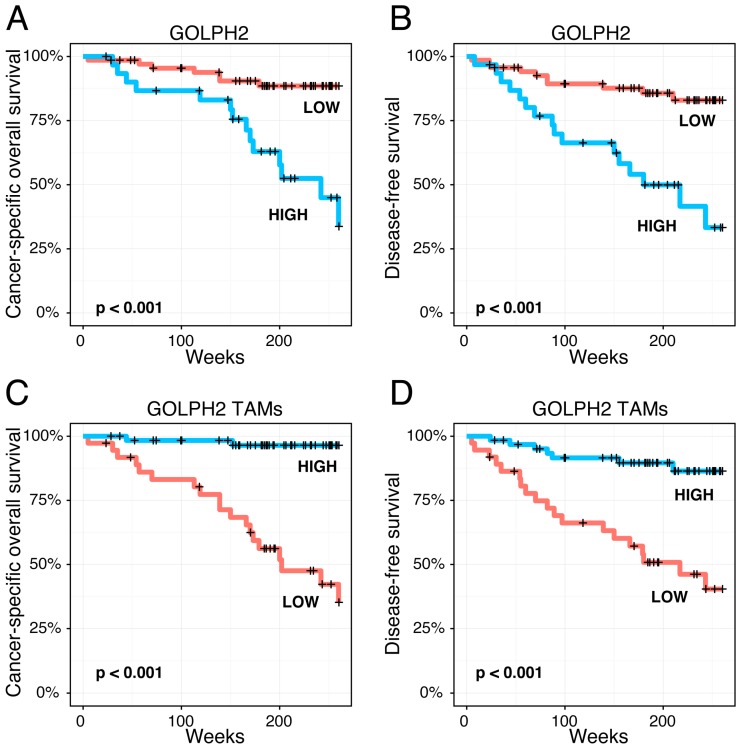
Kaplan-Meier analysis of the prognostic impact of GOLPH2 expression in cutaneous melanoma patients. Enhanced GOLPH2 immunoreactivity in melanoma cells was significantly associated with shorter cancer-specific overall survival (CSOS) and disease-free survival (DFS) (**A** and **B**, respectively); Upregulation of GOLPH2 in tumor-associated macrophages demonstrated contrary prognostic significance and was strongly correlated with longer CSOS and DFS (**C** and **D**, respectively).

**Figure 5:**
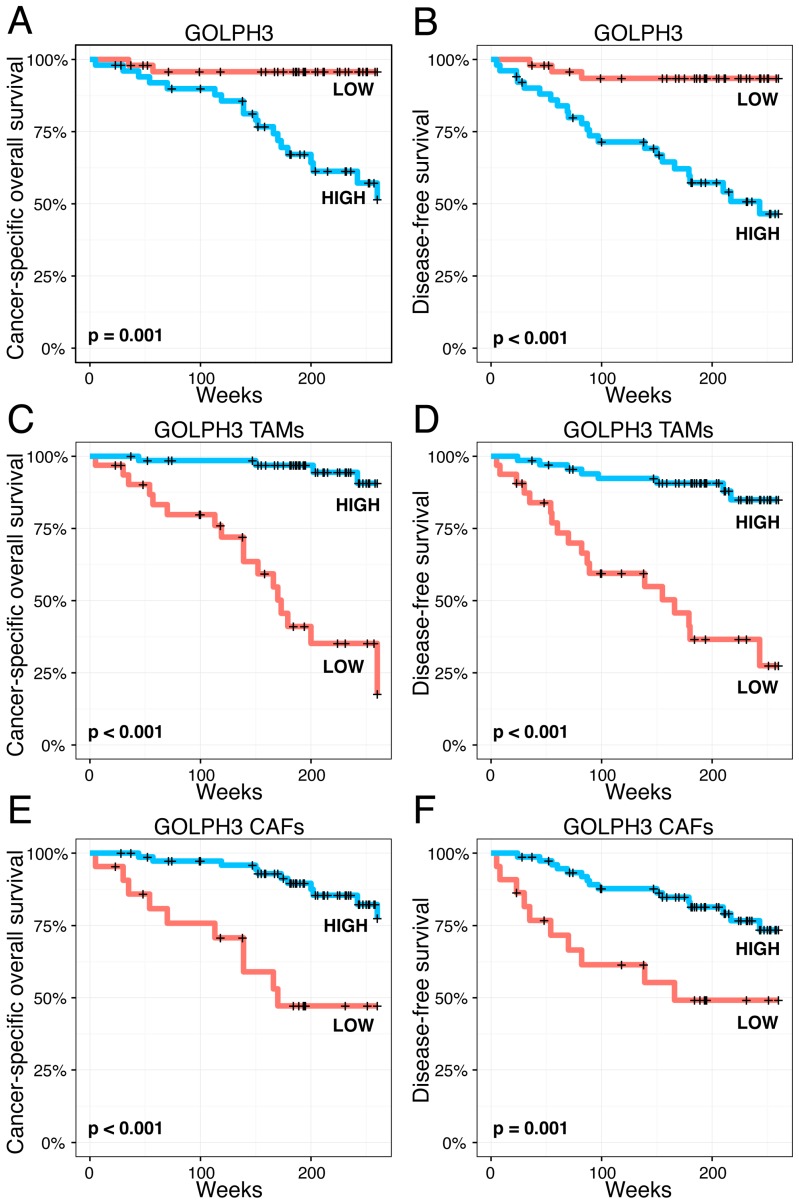
Kaplan-Meier analysis of the prognostic impact of GOLPH3 expression in cutaneous melanoma patients. Upregulation of GOLPH3 in melanoma cells was significantly associated with shorter cancer-specific overall survival (CSOS) and disease-free survival (DFS) (**A** and **B**, respectively). Interestingly, elevated numbers of GOLPH3-positive tumor-associated macrophages and fibroblasts demonstrated contrary prognostic significance and were correlated with significantly longer CSOS and DFS (respectively, **C** and **E** for CSOS, **D** and **F** for DFS).

We revealed that enhanced GOLPH2 and GOLPH3 immunoreactivity in melanoma cells is associated with shorter cancer-specific overall survival (CSOS) and disease-free survival (DFS) in the whole group of patients (Figure 4 and Figure 5). An analogous relation was observed in patients without nodal metastases (data not shown).

Stromal expression of GOLPH2 in TAMs also demonstrated prognostic significance-upregulation of GOLPH2 in TAMs strongly correlated to longer CSOS and DFS both in the whole group of patients (Figure 4) and in a subgroup without nodal metastases (data not shown).

Moreover, Kaplan-Meier analysis showed prognostic significance of GOLPH3 expression in TAMs and CAFs—enhanced immunoreactivity in the stromal compartment of melanoma was associated with prolonged CSOS and DFS, both in the whole study population (Figure 5) and in patients with no nodal metastases (data not shown). GOLPH3 expression in CAFs was an exception not being associated with DFS in patients without nodal metastases (data not shown).

## 3. Discussion

In this study we aimed to examine the expression of GOLPH2 and GOLPH3 in malignant melanocytes and stromal CAFs and TAMs, and to reveal the relationship between these expression levels and other histopathological and clinicopathological parameters. High levels of both proteins in melanoma cells were associated with features of aggressive disease, i.e., greater thickness, ulceration, visible mitotic figures, weak lymphocytic infiltrate (relation with GOLPH3), lymphangioinvasion, nodal metastases and recurrence. Moreover, upregulation of GOLPH2 and GOLPH3 in cancer cells was associated with shorter DFS and CSOS. To the best of our knowledge, this is the first analysis of GOLPH2 and GOLPH3 expression in melanoma in the clinical setting.

Little is known about GOLPH2 functions, but available studies provide some insight into the mechanisms connecting GOLPH2 and carcinogenesis. Jin et al. reported that GOLPH2 promotes HCC invasion by activation of matrix metalloproteinase-13 (MMP-13) [32]. MMP-13 has a documented role in melanoma. Apart from exacerbating melanoma invasiveness [33], it seems to mediate proliferation of cancer cells [34]. In line with in vitro studies, Corte et al. reported a positive correlation between MMP-13 expression and MR in clinical melanoma tissue samples [35]. In their study on gastric cancer, Tang et al. showed that GOLPH2 overexpression attenuated anti-tumor Th1 lymphocyte response [36]. This effect is likely to apply to melanoma, in which Th1-type immune response is a key defensive mechanism.

Recent study by Scott et al. identified GOLPH3 as a potent oncogene amplified in several cancers, including melanoma, which enhances mTOR signaling [26]. mTOR-related pathways control cell growth, proliferation and survival, and are often dysregulated in malignancies [37]. Following studies revealed a number of mechanisms relating GOLPH3 to cancer progression. It mediates formation of metastases by upregulation of MMPs [38,39] and acts as a promotor of angiogenesis [40,41]. Dai et al. showed that GOLPH3 activates NF-κB pathway in HCC [42], while Zeng et al. proved that overexpression of GOLPH3 inhibits transcriptional activity of FOXO1 and, as a consequence, facilitates G1–S-phase transition in breast cancer cells [43]. Both these pathways also play a role in cutaneous melanoma [44,45].

Our results suggest that expression of GOLPH2 and GOLPH3 in TAMs is also clinically relevant for patients with melanoma. We observed increased numbers of GOLPH2-positive and GOLPH3-positive macrophages in thinner, non-ulcerated tumors, with brisk TILs and absent lymphangioinvasion. Distant metastases were not observed among patients with high numbers of GOLPH2-positive TAMs. Increased GOLPH3- and GOLPH2-expressing TAMs were related to prolonged CSOS and DFS. This is the first study that addresses the prognostic significance of GOLPH2 and GOLPH3 expression in TAMs.

TAMs seem to promote melanomagenesis and favor immune escape by their metabolic activity in the local immune milieu [28,46,47]. However, macrophages form a heterogeneous population of cells which dynamically change phenotype during carcinogenesis. The initial response to tumor antigens is mediated by M1 type macrophages, but they often rapidly switch to M2 phenotype under the influence of cytokines secreted by tumor cells [48,49]. Hypoxic conditions emerging within the tumor also stimulate the M1-M2 switch [48]. M1 cells play a great role in melanoma as they promote inflammatory response mediated by TNF-a. Evolution from M1 to M2 phenotype promotes escape of the tumor from immune surveillance [47].

Having no factual insight into the role of GOLPH2 and GOLPH3 in TAMs, we only speculate that the proteins may be associated with prolonged stabilization of M1 phenotype of macrophages, which could result in observed favorable clinical outcome. An indirect indication to an association between activation of local immunity and macrophages expressing GOLPH2 and GOLPH3 is a positive correlation between their number and TIL intensity observed in our study. Careful functional investigations are needed to explore the roles of tumor-associated macrophages in melanoma.

Beneficial influence on survival of high GOLPH3 expression in CAFs is another interesting finding in our results. CAFs are thought to act as promotors of melanoma, but their precise functions and mechanisms of action are not fully understood [50,51,52,53,54]. In our experiment, GOLPH3-expressing fibroblasts were more frequent in thinner, non-ulcerated melanomas, with low mitotic rate and without lymphangioinvasion. Moreover, increased GOLPH3-positive CAFs correlated with absence of regional and distant metastases.

It seems that most of CAFs in melanoma are residual skin fibroblasts which, stimulated by humoral mediators and direct contact with malignant melanocytes, undergo a metabolic transformation to CAFs. This hypothetical process of paraoncogenic transformation of normal fibroblasts to CAFs gradually changes cytobiochemical characteristics of fibroblasts, affecting also their fundamental function, namely the synthesis of extracellular matrix (ECM) components [51,54].

Overexpression of GOLPH3, observed only in some CAFs, might be a feature of initial steps of postulated paraoncogenic transformation in a selected subpopulation of fibroblasts. Stimulation of their metabolism and otherwise quiescent pathway (a manifestation of which would be upregulated GOLPH3) might be an attempt to restrict the expansion of melanoma by increase of ECM production. In such a case, enhanced GOLPH3 immunoreactivity in CAFs might be a potential marker for normal fibroblasts or early stage CAFs, which do not exhibit genetic aberrations or metabolic changes (e.g., increased production of cancer-promoting MMPs) typical for fully developed CAFs [51,54]. The presented interpretation of our results is largely hypothetical due to lack of other studies addressing the role of GOLPH3 in CAFs and no clear criteria discriminating normal fibroblasts and CAFs. The topic warrants further, minute investigations with the use of molecular biology techniques.

To conclude, consistent with findings in other cancer settings, our results suggest a role for GOLPH2 and GOLPH3 in melanoma pathogenesis, but functional studies are needed to evaluate the mechanisms involved.

## 4. Materials and Methods

### 4.1. Patients

100 patients diagnosed with cutaneous melanoma between 2005 and 2010 and receiving treatment in the Lower Silesian Oncology Center in Wroclaw, Poland were enrolled in the study. Enrollment criteria were the availability of tissue material (paraffin blocks and histopathology slides) and medical documentation. Archival medical records were the source of comprehensive clinical data. Information on the diagnostic and therapeutic procedures were retrieved from the medical records at the Lower Silesian Oncology Center‘s oncology outpatient clinic and from the Lower Silesian Cancer Registry and Civil Register Office. The study was approved by the ethical committee of the Wroclaw Medical University, Wrocław, Poland.

Based on the medical records, it was determined that the patients under study were treated by then-available methods and the primary procedure was as follows: the patients underwent excisional biopsy of the primary lesion. When histopathological examination showed cutaneous melanoma, the scar was excised with a margin of 5, 10 or 20 mm of unaffected skin depending on Breslow thickness and primary tumor location, if any. Sentinel lymph node biopsy (SNLB) was performed in patients with no clinically manifest nodal metastases (cN0) and Breslow thickness above 1 mm (>pT1a). Lymphadenectomy was performed when metastases were found (either clinically or by SLNB) in the regional lymph nodes.

The clinical and pathological data of the patients investigated in this study included age and gender, primary tumor location, tumor stratification according to AJCC (pT), presence or absence of nodal (pN) and distant (pM) metastases, information on disease recurrence and SLNB procedures (Table 1 and Table 3).

For tumor samples and histopathological evaluation, archival formalin-fixed and paraffin-embedded tumor specimens were studied. Two pathologists examined all hematoxylin and eosin-stained sections. Breslow thickness, Clark level, growth phase, histologic type, mitotic rate (number of mitotic figures per 1 mm^2^), presence of ulceration, lymphangioinvasion, microsatellitosis, intensity of TILs and microscopic evidence of regression (Table 2 and Table 4) were recorded in pathology reports for the primary tumor.

The semi-quantitative method described below was used to assess TILs. Absence of TILs: there are no lymphocytes present or lymphocytes are present but they do not infiltrate tumor at all. Non-brisk TILs: lymphocytes infiltrate melanoma only focally or not along the entire base of the vertical growth phase. Brisk TILs: lymphocytes diffusely infiltrate the entire base of the vertical growth phase or the entire invasive component of the melanoma.

### 4.2. Immunohistochemistry

GOLPH2 and GOLPH3 immunoreactivity was analyzed in 20 normal skin specimens (sampled during autopsy or non-oncological procedures), 30 benign nevi and 100 cutaneous melanoma patients. Immunohistochemical evaluation of GOLPH2 expression (mouse monoclonal antibody, clone F-2, sc-365817; dilution: 1:100; Santa Cruz Biotechnology, Dallas, TX, USA) and GOLPH3 (rabbit polyclonal antibody, LS-B5044; dilution 1:1000; LifeSpan Biosciences, Seattle, WA, USA) was performed on 4 µm-thick paraffin sections mounted on sialinized slides (code number S 3003; DAKO, Glostrup, Denmark), which were then subjected to deparaffinization, rehydration and heat-induced epitope unmasking, performed using PT Link, with EnVisionTM Target Retrieval Solution used for 20–40 min incubation at 97 °C. Autostainer Link was used to perform immunological test using detection reagents DakoEnVisionTMFLEX/HRP (SM802).

To visualize TAMs and CAFs, slides were stained immunohistochemically with anti-CD68 (monoclonal mouse antibody, clone KP1, nr IS 609 RTU*FLEX, dilution by the producer, DAKO) and anti-SMA (monoclonal mouse antibody, clone 1A4, nr IS 611 RTU*FLEX, dilution by the producer, DAKO) antibodies and anti-Melan-A antibody (mouse monoclonal antibody, clone A103, nr IS 633 RTU*FLEX, dilution by the producer, DAKO) as a control reaction.

Cells positively stained for CD68 and negative for SMA and Melan-A were regarded as macrophages, whereas cells positive for SMA and negative for CD68 and Melan-A were regarded as fibroblasts. Besides immunohistochemical profile, visual assessment of cell cytomorphology was the basis of the final interpretation of microscopic image.

Only an overlap between immunohistochemical (IHC) profile and adequate, non-malignant morphology of cells (small, normochromatic nuclei, no aberrations of nuclear shape, absent nucleoli, proper nuclear/cytoplasmic ratio) qualified them as macrophages or fibroblasts.

In interpretation of IHC reactions, to avoid errors related to the use of brown chromogen to visualize expression of GOLPH2 and GOLPH3 in TAMs (which may contain melanin, imitating a positive reaction), control IHC reactions were performed in Autostainer Link using detection reagents EnVisionTM G/2 System/AP Rabbit/Mouse/Permanent Red (nr K 5355, DAKO) with red chromogen.

### 4.3. Evaluation of Immunohistochemistry

Two independent pathologists evaluated the IHC reaction intensity. In case of doubt, the samples were re-evaluated under a double-headed microscope and the result was discussed until final agreement.

The expression of GOLPH2 was observed in two cell populations: melanoma cells and TAMs. GOLPH3 immunoreactivity was observed in three compartments: melanoma cells, TAMs and CAFs. For every cell type, an individual system of IHC reaction assessment was used.

A semi-quantitative method was used to evaluate GOLPH2 and GOLPH3 expression in melanoma cells. The two immunohistochemical reaction parameters used were the percentage of cells with a positive cytoplasmic reaction (the percentage of reactive tissue) and the intensity of reaction. The semi-quantitative IRS (ImmunoReactive Score) scale of Remmele and Stenger [55] was used to calculate the final immunohistochemical reaction results. In this approach, points are given depending on the percentage of reactive cells (0–4 points) and intensity of reaction (0–3 points). The scores for these two parameters are multiplied to give the final result referred to as IRS factor or score (0–12 points).

GOLPH2 and GOLPH3 immunoreactivity in TAMs and CAFs was evaluated in five hot-spots characterized by the highest intensity of a corresponding IHC reaction at 400× magnification. A mean number of GOLPH2- and GOLPH3-positive cells was calculated in each case. Final interpretation of IHC reaction with anti-GOLPH2 and anti-GOLPH3 antibodies included three other microscopic parameters, i.e., cyto- and immunopathological features of macrophage or fibroblast phenotype: (1) non-malignant morphology of cells (small, normochromatic nuclei, no aberrations of nuclear shape, absent nucleoli, proper nuclear/cytoplasmic ratio); (2) positive anti-CD68 or anti-SMA stain; and (3) lack of Melan-A positivity.

### 4.4. Statistical Analysis

Statistical analysis was performed using the R language (Available online: https://www.r-project.org/). Continuous variables like the age or proportions of lymphocytes were summarized with the use of the mean, median, min and max values. The expression of analyzed proteins in evaluated compartments was divided into two levels based on IRS score for melanoma cells (<6 vs. ≥6), the number of GOLPH2- and GOLPH3-positive macrophages (<20 vs. ≥20) and the number of GOLPH3-positive fibroblasts (<10 vs. ≥10). For CSOS DFS, we performed log-tests and Kaplan-Meier curves; all such analyses were conducted with the survival package for R. To assess the relation between dichotomized GOLPH2 and GOLPH3 expression in melanoma cells, TAMs and CAFs, and continuous variables, the Wilcoxon two-sample test was used. The relation of their immunoreactivity with binary variables was assessed by Fisher’s exact test while the relation with other categorical variables was assessed by chi-square test. All relations were summarized by a suitable p-value, and all p-values smaller than 0.05 were considered as significant.

## 5. Conclusions

GOLPH2 and GOLPH3 are Golgi-related proteins associated with aggressiveness and progression of a number of cancers, but their prognostic significance in melanoma has not been investigated. Using immunohistochemical stainings, we analyzed GOLPH2 and GOLPH3 expression in cancer cells, TAMs and CAFs in 100 primary melanoma tissue samples. To the best of our knowledge, this is the first analysis of GOLPH2 and GOLPH3 expression in clinical melanoma specimens, and the first one addressing the significance of their expression in the stromal components. We revealed that high levels of both proteins in melanoma cells were associated with characteristics of aggressive disease, and shorter DFS and CSOS. On the contrary, increased numbers of GOLPH3-positive CAFs and TAMs, and GOLPH2-positive TAMs were more frequently observed in less aggressive tumors, and were related to longer DFS and CSOS. GOLPH2 expression was not observed in CAFs. Our results suggest that GOLPH2 and GOLPH3 play a role in melanoma progression and are potential targets for molecular-based therapies. We report for the first time the significance, contrary to that in cancer cells, of GOLPH2 and GOLPH3 expression in stromal TAMs and CAFs.

## Figures and Tables

**Table 1:** Correlations between GOLPH2 immunoreactivity in melanoma cells and tumor-associated macrophages, and clinical parameters.

Clinical Parameters	GOLPH2 Immunoreactivity					
	Melanoma Cells			Tumor-Associated Macrophages (TAMs)		
	Low	High	*p* Value	Low	High	*p* Value
**Age in years (21–79) ^a^**						
mean, 56 ± 15.4; median, 58			1.000			0.555
**Gender ^b^**						
Female	44	15	0.220	26	33	0.122
Male	25	16		11	30	
**Primary tumor location ^c^**						
Head/neck	10	4	0.911	9	5	0.145
Extremities	31	12		13	30	
Hand/foot	2	1		1	2	
Trunk	26	14		14	26	
**Primary tumor (pT) ^a^**						
pT1	30	4	**0.002**	5	29	**0.005**
pT2	16	5		10	11	
pT3	10	13		9	14	
pT4	13	9		13	9	
**Regional lymph nodes status (pN) ^b^**						
No metastases (pN−)	64	21	**0.003**	29	56	0.258
Metastases present (pN+)	5	10		8	7	
**Distant metastases (pM) ^b^**						
No metastases (pM−)	68	27	0.053	32	63	**0.012**
Metastases present (pM+)	1	4		5	0	
**Sentinel lymph node biopsy status (SNLB) ^b^ (57 patients)**						
No metastases (SNLB−)	37	10	**0.007**	10	37	0.398
Metastases present (SNLB+)	3	7		4	6	
**Recurrence ^b^**						
No	63	21	**0.007**	27	57	**0.043**
Yes	6	10		10	6	

^a^ *p* value of Wilcoxon two-sample test; ^b^ *p* value of Fisher’s exact test; ^c^ *p* value of chi^2^ test; statistically significant results (*p* < 0.05) are in bold text.

**Table 2:** Correlations between GOLPH2 immunoreactivity in melanoma cells and tumor-associated macrophages, and pathological parameters.

Histopathological Parameters	GOLPH2 Immunoreactivity					
	Melanoma Cells			Tumor-Associated Macrophages (TAMs)		
	Low	High	*p* Value	Low	High	*p* Value
**Breslow thickness ^a^**						
≤1 mm	30	4	**0.004**	5	29	**0.004**
1.01–2.00 mm	15	5		10	10	
2.01–4.00 mm	11	13		9	15	
>4 mm	13	9		13	9	
**Clark level ^a^**						
I	-	-	**0.004**	-	-	**0.015**
II	17	1		3	15	
III	35	13		16	32	
IV	14	11		11	14	
V	3	6		7	2	
**Histologic type ^b^**						
Superficial spreading melanoma (SSM)	47	20	0.939	23	44	0.691
Nodular malignant melanoma (NMM)	20	10		13	17	
Acral-lentiginous melanoma (ALM)	2	1		1	2	
**Mitotic rate ^a^**						
0	37	8	**0.019**	12	33	0.138
≥1	32	23		25	30	
**Ulceration ^c^**						
No	45	10	**0.005**	15	40	**0.043**
Yes	24	21		22	23	
**TILs ^c^**						
No	10	8	0.105	11	7	**0.038**
Non-brisk	19	12		12	19	
Brisk	40	11		14	37	
**Microsatellitosis ^c^**						
No	65	30	0.960	34	61	0.537
Yes	4	1		3	2	
**Lymphatic invasion ^c^**						
No	58	15	**<0.001**	19	54	**<0.001**
Yes	11	16		18	9	
**Tumor regression ^c^**						
No	64	28	0.987	35	57	0.725
Yes	5	3		2	6	

^a^ *p* value of Wilcoxon two-sample test; ^b^ *p* value of chi^2^ test; ^c^ *p* value of Fisher’s exact test; statistically significant results (*p* < 0.05) are in bold text.

**Table 3:** Correlations between GOLPH3 immunoreactivity in melanoma cells, tumor-associated macrophages and cancer-associated fibroblasts, and clinical parameters.

Clinical Parameters	GOLPH3 Immunoreactivity								
	Melanoma Cells			Tumor-Associated Macrophages (TAMs)			Cancer-Associated Fibroblasts (CAFs)		
	Low	High	*p* Value	Low	High	*p* Value	Low	High	*p* Value
**Age in years (21–79) ^a^**									
mean, 56 ± 15.4; median, 58			1.000			0.910			0.984
Gender ^b^									
Female	31	28	0.518	18	41	0.868	16	43	0.216
Male	18	23		14	27		6	35	
**Primary tumor location ^c^**									
Head/neck	8	6	0.338	9	5	**0.017**	5	9	0.160
Extremities	22	21		10	33		5	38	
Hand/foot	0	3		2	1		1	2	
Trunk	19	21		11	29		11	29	
**Primary tumor (pT) ^a^**									
pT1	25	9	**0.001**	1	33	**<0.001**	1	33	**0.005**
pT2	11	10		10	11		5	16	
pT3	8	15		11	12		7	16	
pT4	5	17		10	12		9	13	
**Regional lymph nodes status (pN) ^b^**									
No metastases (pN−)	45	40	0.110	25	60	0.307	15	70	**0.031**
Metastases present (pN+)	4	11		7	8		7	8	
**Distant metastases (pM) ^b^**									
No metastases (pM−)	48	47	0.383	28	67	0.062	18	77	**0.008**
Metastases present (pM+)	1	4		4	1		4	1	
**Sentinel lymph node biopsy status (SNLB) ^b^ (57 patients)**									
No metastases (SNLB−)	27	20	**0.018**	10	37	0.398	4	43	0.177
Metastases present (SNLB+)	1	9		4	6		3	7	
**Recurrence ^b^**									
No	47	37	**0.004**	22	62	**0.010**	19	65	0.989
Yes	2	14		10	6		3	13	

^a^ *p* value of Wilcoxon two-sample test; ^b^ *p* value of Fisher’s exact test; ^c^ *p* value of chi^2^ test; statistically significant results (*p* < 0.05) are in bold text.

**Table 4:** Correlations between GOLPH3 immunoreactivity in melanoma cells, tumor-associated macrophages and cancer-associated fibroblasts, and pathological parameters.

Histopathological Parameters	GOLPH3 Immunoreactivity								
	Melanoma Cells			Tumor-Associated Macrophages (TAMs)			Cancer-Associated Fibroblasts (CAFs)		
	Low	High	*p* Value	Low	High	*p* Value	Low	High	*p* Value
**Breslow thickness ^a^**									
≤1 mm	25	9	**<0.001**	1	33	**<0.001**	1	33	**0.006**
1.01−2.00 mm	11	9		9	11		5	15	
2.01−4.00 mm	8	16		12	12		7	17	
>4 mm	5	17		10	12		9	13	
**Clark level ^a^**									
I	-	-	**0.001**	-	-	**0.021**	-	-	**0.012**
II	16	2		1	17		1	17	
III	21	27		15	33		8	40	
IV	10	15		11	14		8	17	
V	2	7		5	4		5	4	
**Histologic type ^b^**			**0.042**			**0.036**			
Superficial spreading melanoma (SSM)	38	29		16	51		12	55	0.369
Nodular malignant melanoma (NMM)	11	19		14	16		9	21	
Acral-lentiginous melanoma (ALM)	0	3		2	1		1	2	
**Mitotic rate ^a^**									
0	31	14	**0.002**	9	36	**0.037**	4	41	**0.010**
≥1	18	37		23	32		18	37	
**Ulceration ^c^**									
No	35	20	**0.002**	10	45	**0.002**	6	49	**0.007**
Yes	14	31		22	23		16	29	
**TILs ^c^**									
No	4	14	**0.017**	12	6	**<0.001**	7	11	0.071
Non-brisk	14	17		11	20		8	23	
Brisk	31	20		9	42		7	44	
**Microsatellitosis ^c^**									
No	47	48	1.000	29	66	0.376	20	75	0.658
Yes	2	3		3	2		2	3	
**Lymphatic invasion ^c^**									
No	44	29	**<0.001**	18	55	**0.019**	10	63	**0.003**
Yes	5	22		14	13		12	15	
**Tumor regression ^c^**									
No	46	46	0.757	28	64	0.458	20	72	1.000
Yes	3	5		4	4		2	6	

^a^ *p* value of Wilcoxon two-sample test; ^b^ *p* value of chi^2^ test; ^c^ *p* value of Fisher’s exact test; statistically significant results (*p* < 0.05) are in bold text.
